# Egg Case Protein 3: A Constituent of Black Widow Spider Tubuliform Silk

**DOI:** 10.3390/molecules26165088

**Published:** 2021-08-22

**Authors:** Mikayla Shanafelt, Camille Larracas, Simmone Dyrness, Ryan Hekman, Coby La Mattina-Hawkins, Taylor Rabara, Wilson Wu, Craig A. Vierra

**Affiliations:** 1 Departments of Chemistry and Biological Sciences, University of the Pacific, Stockton, CA 95211, USA; shanafeltm1@gmail.com (M.S.); c_larracas@u.pacific.edu (C.L.); simmonedyrness@gmail.com (S.D.); Cobylamattina@gmail.com (C.L.M.-H.); Taylor.rabara@vaxcyte.com (T.R.); w_wu15@u.pacific.edu (W.W.); 2 Center for Network Systems Biology, Boston University, Boston, MA 02215, USA; rhekman@bu.edu

**Keywords:** spider silk, spidroins, tubuliform silk, black widow spider

## Abstract

Spider silk has outstanding mechanical properties, rivaling some of the best materials on the planet. Biochemical analyses of tubuliform silk have led to the identification of TuSp1, egg case protein 1, and egg case protein 2. TuSp1 belongs to the spidroin superfamily, containing a non-repetitive *N*- and *C*-terminal domain and internal block repeats. ECP1 and ECP2, which lack internal block repeats and sequence similarities to the highly conserved *N*- and *C*-terminal domains of spidroins, have cysteine-rich *N*-terminal domains. In this study, we performed an in-depth proteomic analysis of tubuliform glands, spinning dope, and egg sacs, which led to the identification of a novel molecular constituent of black widow tubuliform silk, referred to as egg case protein 3 or ECP3. Analysis of the translated ECP3 cDNA predicts a low molecular weight protein of 11.8 kDa. Real-time reverse transcription–quantitative PCR analysis performed with different silk-producing glands revealed ECP3 mRNA is predominantly expressed within tubuliform glands of spiders. Taken together, these findings reveal a novel protein that is secreted into black widow spider tubuliform silk.

## 1. Introduction

Spider silk is a high-performance fiber renowned for its extraordinary mechanical properties, rivaling steel, Kevlar, and a wide range of other natural and manmade materials [1,2]. The western black widow, *Latrodectus hesperus*, has become one of the most extensively studied spider species with respect to silk gene expression and fiber constituents, both at the level of transcriptomics and proteomics [3,4,5,6,7,8,9,10,11]. Black widow spiders, which are cobweavers, are members of the superfamily Araneoidea. Morphological studies of female black widow spiders reveal seven distinct silk-producing abdominal glands, including the major ampullate, minor ampullate, tubuliform, aciniform, pyriform, aggregate, and flagelliform glands [12,13]. Each gland produces fibers or glues with distinct mechanical and biomolecular properties that are highly tuned to specific biological functions [14]. Differences in the mechanical properties of the threads are largely due to altered protein compositions, which are strongly correlated with differential expression of structural proteins called spidroins, a contraction of the words spider and fibroin [15,16]. Spidroins, therefore, represent a family of spider-specific fibroins that have significant roles in fiber mechanics. Members of this family have common biochemical characteristics, including large molecular masses with internal block repeats and non-repetitive *N*- and *C*-terminal domains within their protein sequences [16,17,18,19]. Although the spidroin family protein architectures all share internal block repeats, their specific amino acid sequences and lengths differ, and these differences in primary amino acid sequences have been a focal point of the scientific community to establish relationships between protein sequence, structure, and fiber mechanics [20,21,22].

The major ampullate and tubuliform glands have different morphological features, but both lumens store a liquid spinning dope that is extruded into a dry fiber via external spigots on the spider’s spinnerets. Despite morphological distinctions, these two glands are reported to share similar spidroin gene expression profiles [3]. In black widow spiders, the major ampullate gland extrudes dragline silk (also called major ampullate silk or MA silk), a fiber type used for locomotion and web construction, whereas the tubuliform gland manufactures the main constituent of egg cases, tubuliform silk. Egg cases, which also contain aciniform silk, enclose eggs and protect them from predators and environmental fluctuations, such as changes in temperature and humidity [23]. Tubuliform silk has been shown to contain the spidroin TuSp1, along with two non-spidroin proteins called egg case protein 1 and 2 (ECP1 and ECP2) [6,24,25,26]. Additionally, a recent proteomic analysis of tubuliform glands, along with deep transcriptome sequencing studies to identify silk-gland specific transcripts (SSTs), has led to the discovery of 14 SST proteins in tubuliform glands of black widow spiders [5]. Two of these 14 SST proteins have been previously shown to be constituents of tubuliform silk, which include ECP1 and TuSp1 [6,24,25,26]. However, it is unclear whether the other 12 SST proteins are extruded from the tubuliform gland, or alternatively, whether these factors represent non-extruded proteins that participate in the spider silk assembly pathway. To further emphasize the importance of this work, the identification of novel constituents of spider silk, specifically tubuliform silk, has significant value for the spider silk community, as insight into the ingredients that comprise natural silk has direct implications on synthetic silk production and its potential use for military, industrial, and engineering applications.

In this study, we performed the most comprehensive proteomic analysis to date of tubuliform glands, its liquid spinning dope, and spun egg cases to provide additional insight into the molecular constituents of tubuliform silk, the dominant material of egg sacs. We hypothesized that proteins detected in all three structures (tubuliform glands, liquid spinning dope, and egg cases) represent excellent candidates for molecular components of tubuliform silk. 

## 2. Results and Discussion

### 2.1. Proteomic Analysis of Tubuliform Glands, Spinning Dope, and Egg Sacs

**Figure 1:**
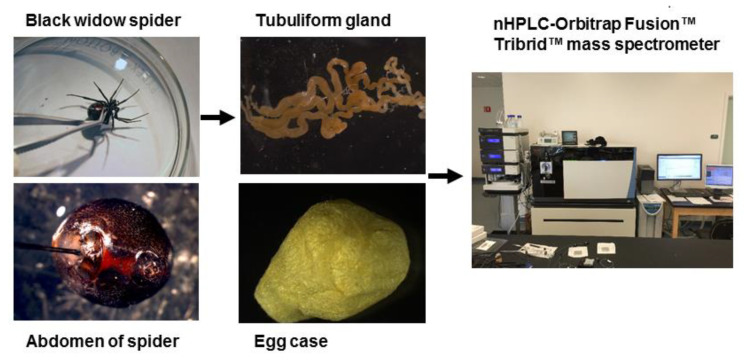
General workflow for isolation of the tubuliform gland, egg sac, and dope (liquid removed from the tubuliform glands), followed by analysis of the tryptic peptides by MS/MS analysis.

**Figure 2:**
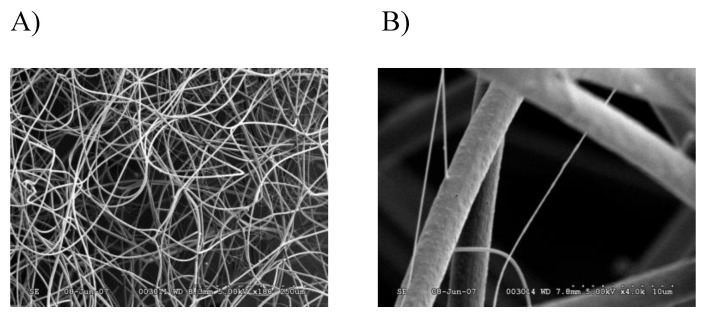
SEM images of black widow spider egg case silk reveal two different diameter fibers: (**A**) 180× magnification; (**B**) 4000× magnification.

In order to perform a comprehensive, in-depth analysis of the constituents of tubuliform silk, we developed a generalized proteomic workflow designed to analyze proteins expressed in tubuliform glands, the spinning dope, which represents the liquid components within the lumen of the glands and the egg sacs of black widow spiders using mass spectrometry (Figure 1). Scanning electron microscope studies of black widow spider egg cases have previously revealed two distinct fiber types [25]. The diameter sizes are approximately 5 μm and 0.5 μm, corresponding to tubuliform and aciniform silk, respectively, with the majority of the volume comprising tubuliform silk (Figure 2A,B).

We collected egg sacs from black widow spiders, dissolved the fibers in chaotropic solvents, and performed in solution tryptic digestion, followed by nLC-MS/MS analysis. Analysis of the egg cases led to the identification of 30 protein groups, 174 peptide groups, 523 peptide spectral matches (PSMs), and 30,985 MS^2^ spectra (File S1). After manual inspection, the number of protein groups was reduced from 30 to 25 due to protein duplications entered into the database with different accession codes. The number of unique peptides that mapped back to targets ranged from 1 to 34. Some of the proteins found within egg cases included ECP1, ECP2, TuSp1, AcSp1, MaSp1, MaSp2, Flagelliform spidroin variant 1, aggregate spidroin variant 1, PySp1, cysteine-rich protein 2 (CRP2), and CRP4 (File S1). ECP1, ECP2, and TuSp1 have previously been shown to represent constituents of tubuliform silk, while AcSp1 has been shown to be components of dragline, wrapping, and egg case silk [5,6,8,24,26]. Moreover, both MaSp1 and MaSp2 mRNAs have been shown to be present within tubuliform glands, suggesting tubuliform silk represents a composite of spidroins. The detection of aggregate spidroin variant 1 in egg sacs also supports the presence of glue proteins extruded from the aggregate gland during the spinning of egg cases, while the presence of PySp1 may indicate attachment disc silk is used to help immobilize the egg cases to a solid support. 

**Figure 3:**
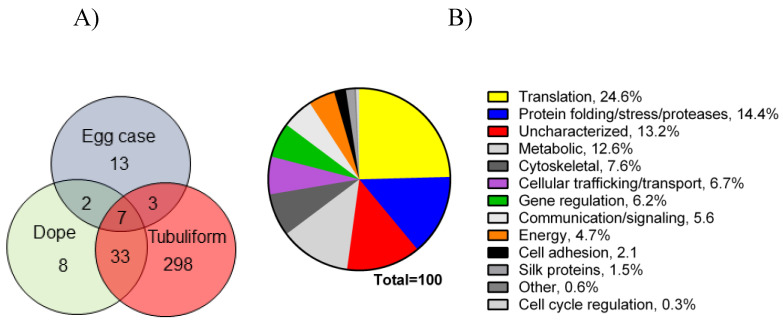
Venn diagram representing number of proteins expressed in tubuliform glands, liquid spinning dope, and egg sacs, along with a pie chart of percentages of proteins expressed in the tubuliform gland categorized into several different molecular and cellular functions: (**A**) Venn diagram; (**B**) pie chart.

Following the spinning of the egg sacs by female black widow spiders, we removed their tubuliform glands by microdissection, dissolved the glands in HFIP, and then performed in-solution tryptic digestion, followed by MS/MS analysis (Figure 1). Prior to lysis, we removed the spinning dope from the tubuliform glands, which was performed by the collection of internal liquid contents from the tubuliform glands. Analysis of the tubuliform glands led to the identification of 409 protein groups (collapsed to 341 protein groups; Figure 3A), 1118 peptide groups, 1832 PSMs, and 15,777 MS^2^ spectra (File S2). Consistent with previous studies, ECP1, ECP2, and TuSp1 were expressed in tubuliform glands. CRP4, a CRP family member, was also demonstrated to exist for the first time at the protein level within tubuliform glands. Although CRP4 was expressed in tubuliform glands, this observation differs from a previous transcriptomic analysis of *L. hesperus*, in which CRP1 and CRP3 mRNAs were detected in tubuliform glands [27]. Other proteins involved in a wide range of molecular and cellular functions were identified, including translation, protein folding and processing, cellular communication (signaling), metabolism, cytoskeletal activities, cell adhesion, and gene regulation (Figure 3B).

Translation, protein folding and processing, and enzymes involved in metabolic reactions constituted approximately 25%, 14%, and 13%, respectively, of the proteome (Figure 3B). Some of the proteins expressed in tubuliform glands represented uncharacterized factors, while others fell into a broad range of cellular activities (Figure 3B). Overall, these data support that a large number of tubuliform gland proteins participate in silk protein biosynthesis. 

**Figure 4:**
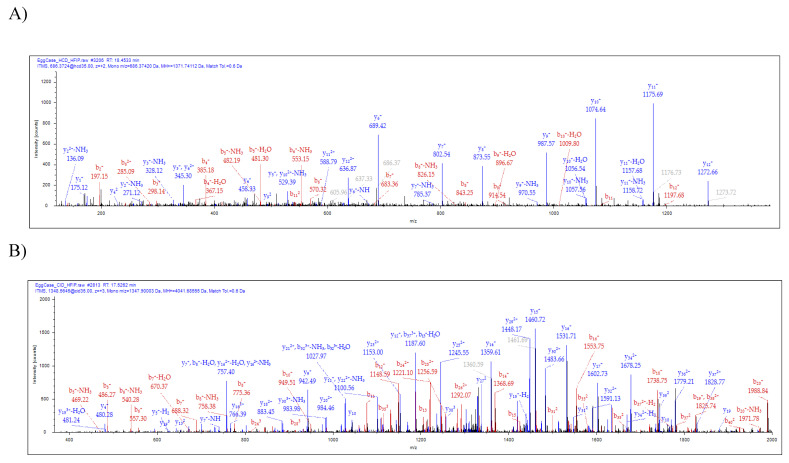
Tandem MS analysis of tryptic fragments generated from in-solution digestion of tubuliform spinning dope identifies the presence of a CRP4 peptide sequence, VVGPFPICDYGLR, and peptide sequence of an uncharacterized protein, SQGNVAMSSSQAGYG QGQSYSSNYAATGDSGTGQGGYSSMR. (**A**) HCD (MS/MS) spectrum of CRP4 precursor ion with MH^+^ *m/z* 1271.74 supports the presence of CRP4 in spinning dope; (**B**) CID (MS/MS) spectrum of the uncharacterized protein’s precursor ion with MH^+^ *m/z* 4041.68 supports the presence of ECP3 in spinning dope. Y- and b-ions and their corresponding masses are shown above the peaks.

Analysis of the spinning dope revealed 50 different protein groups. ECP1, ECP2, MaSp1, MaSp2, TuSp1, along with CRP4, were identified in the dope (File S3). Comparison of our proteomic data from egg cases, tubuliform glands, and spinning dope revealed seven proteins common to all three locations, which included TuSp1 (egg case fibroin), ECP1, ECP2, fasciclin, CRP4, and two uncharacterized proteins (Figure 3A). Representative MS/MS spectra corresponding to CRP4 and one of the uncharacterized proteins are shown (Figure 4A,B). The discovery of a common group of seven proteins in the liquid dope and egg cases further substantiates the assertion that these molecules are constituents of tubuliform silk. Moreover, it implies CRP4, which has been shown to be a constituent of dragline silk, may have a generalized functional role in both fiber types [7]. 

Previous studies have identified 14 SST proteins in tubuliform glands [5]. Of the 14 SST proteins, 8 were shown to be expressed exclusively in tubuliform glands; these eight SST proteins were not detected in the major or minor ampullate glands. Of the eight SST tubuliform gland proteins, two were known constituents of tubuliform silk (ECP1 and TuSp1), while the other six SST proteins were classified as uncharacterized proteins [5]. In order to determine whether these six SSTs were present in tubuliform spinning dope and egg cases, we analyzed a customized protein database containing only these six translated SSTs. Two of the SST proteins were detected in both locations (dope and egg cases), which corresponded to transcriptome scaffold ID numbers 129 (silk_comp16922_c0_seq1) and 503 (Contig242) (File S4). One of the SST proteins, transcriptome scaffold ID #717 (silk_Contig894), was found exclusively in tubuliform glands, while the remaining three SST proteins were not found in either tubuliform glands, dope, or egg case fibers.

### 2.2. Characterization of a Novel Protein Identified in Tubuliform Silk

**Figure 5:**
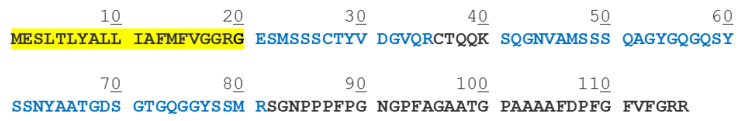
Translation of the gene sequence of GenBank accession number HQ005929.1 predicts a low molecular weight protein with 116 amino acids. Blue text corresponds to peptide sequences identified by MS/MS studies. Yellow coloration denotes a putative secretion signal sequence identified by the SignalP-5.0 server [28].

To further characterize transcriptome scaffold ID #503 (GenBank accession number HQ005929.1 or UniProt Accession E7D1H9) we inspected the translated cDNA, which revealed an open reading frame (ORF) with a methionine translational start codon and an in-frame stop codon. Amino acid composition analysis of this protein revealed the highest percentages of amino acids Ala, Gly, and Ser, which were present in 11.2%, 17.2%, and 13.8%, respectively. The predicted molecular mass of the silk protein was 11.8 kDa, with a pI = 7.76 (Figure 5).

As silk proteins contain secretion signals on their *N*-termini, we inspected the translated cDNA sequence with SignalP-5.0 for the presence of a putative secretion signal. Consistent with secreted proteins, SignalP-5.0 predicted a likelihood of a signal peptide at 0.9972 probability, with a cleavage site between residues 20 to 21 at 61.9% (GRG-ES) (Figure 5). Analysis of our MS/MS data from in-solution tryptic digestion of the tubuliform spinning dope and egg cases detected two distinct tryptic cleavage products that mapped to the uncharacterized protein, translated transcriptome scaffold ID #503 (Figure 4B and Figure 5). These peptides did not include the predicted secretion signal, supporting the assertion that this region was removed by a signal peptidase prior to extrusion. Analysis of its primary sequence by PHYRE2, a protein structure prediction algorithm, supported this protein contains significant regions of intrinsic disorder [29]. Collectively, given the discovery of this uncharacterized protein in tubuliform glands, spinning dope, and egg cases by MS/MS analysis, we have named this protein egg case protein 3 (ECP3).

**Figure 6:**
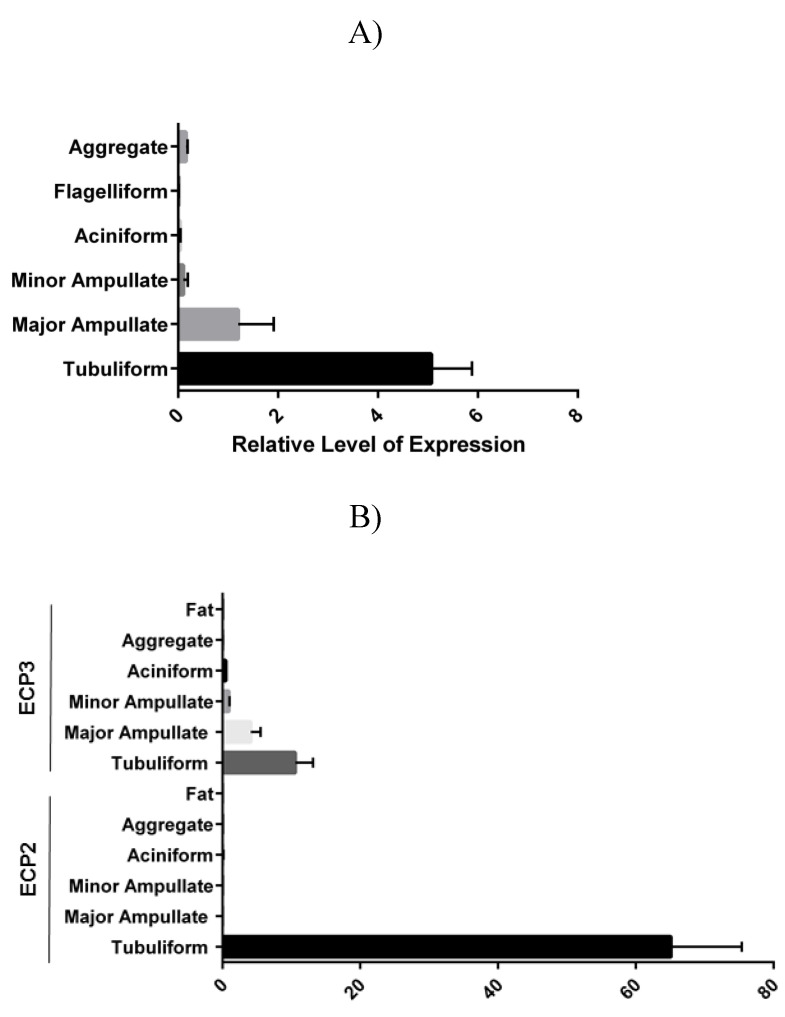
ECP3 mRNA is expressed in the highest levels in the tubuliform gland but has lower levels relative to ECP2 mRNA: (**A**) real-time RT-PCR analysis of ECP3 transcript levels in six different silk-producing glands; (**B**) real-time RT-PCR analysis of ECP2 and ECP3 mRNA levels in the five different silk-producing glands and fat tissue.

To investigate the expression pattern of ECP3 in spider tissues, we performed real-time RT-qPCR analysis. The abdomens of female black widow spiders were subject to microdissection to remove the silk-producing glands (major and minor ampullate, tubuliform, flagelliform, aggregate, and aciniform glands). Analysis of ECP3 mRNA expression profiles revealed the highest levels in tubuliform glands (Figure 6A). Essentially, no ECP3 transcripts were detected in the aggregate and flagelliform glands, whereas low levels were observed in the major ampullate, minor ampullate, and aciniform glands (Figure 6A). ECP3 mRNA expression levels were greater than twofold higher in tubuliform glands, compared to major ampullate glands (Figure 6A). Additionally, no ECP3 mRNA expression was detected in fat tissue (Figure 6B).

Intriguingly, ECP3 mRNA was detected in the major ampullate gland, albeit at levels lower relative to the tubuliform gland, suggesting ECP3 also plays a role in major ampullate silk. This observation is consistent with a proteomic analysis of dragline silk performed by our laboratory and its presence as an uncharacterized protein [7].

Previously, we have demonstrated ECP2 mRNA is highly expressed in tubuliform glands [6]. To compare the relative levels of ECP2 to ECP3 mRNA levels, we compared their mRNA expression profiles. Relative to ECP2 mRNA expression, ECP3 mRNA levels were approximately 3.5-fold lower (Figure 6B). Taken together, these data support ECP3 mRNA levels, albeit lower relative to ECP2 mRNA, are expressed at considerable levels in tubuliform glands.

## 3. Materials and Methods

### 3.1. Spider Care

Adult female *Latrodectus hesperus* spiders were collected in San Joaquin County, CA. Spiders were housed in square plastic cages and maintained on a regular diet of crickets. Prior to dissection of the tubuliform glands, spiders were anesthetized with CO_2_ gas, followed by severing of the cephalothorax at the midpoint. Silk-producing glands were dissected from the abdomen submerged under a Ringer’s solution (0.15 M sodium chloride, 0.015 M sodium citrate). Removal of silk-producing glands was monitored using a Leica MZ 16 stereomicroscope, and images were collected using a Leica MC 170 HD digital camera. For proteomic studies, tubuliform glands were removed from 3 different female black widow spiders immediately after egg sacs were spun. Tubuliform spinning dope was removed by carefully pressing on the exterior of the glands with a blunt pair of forceps.

### 3.2. Sample Preparation for Proteomics

Tubuliform glands, egg sacs, and spinning dope proteins were dissolved in 100% (*v*/*v*) Hexafluoro-2-propanol (HFIP), then subject to flash freeze in liquid nitrogen, lyophilized, then redissolved in 8 M GdnHCl. Dissolved tissues were reduced by the addition of 5 mM dithiothreitol (DTT) for 30 min at 55 °C. After reduction, the samples were allowed to come to room temperature, followed by alkylation of reduced sulfhydryl groups via the addition of 15 mM iodoacetamide (IAA) for 30 min. The alkylation reactions were quenched by the addition of 5 mM DTT. The samples were then diluted with water to reduce the concentration of 8 M GdnHCl to below 1 M, then proteolytic digestions were performed by the addition of trypsin (Promega, Madison, WI, USA), 1:25 enzyme to protein ratio. Samples were digested overnight at 37 °C; then the next morning, additional trypsin was added and samples were incubated for an additional 2 hr at 37 °C. Digestions were terminated via acidification by the addition of Trifluoroacetic acid (TFA) and desalted over Pierce™ *C*-18 spin columns (Thermo Fisher Scientific, Waltham, MA, USA).

### 3.3. nLC-ESI-MS/MS Analysis

Nanoscale liquid chromatography–tandem mass spectrometry was performed using an Orbitrap Fusion™ Tribrid™ mass spectrometer (Thermo Fisher Scientific) operated in a data-dependent manner by Xcalibur 4.0 software. Eluting peptides were converted from liquid to gas-phase cations by electrospray ionization using an Easy-Spray™ ion source. MS^1^ survey scans of precursor ions from 200 to 1400 *m/z* were performed using the orbitrap mass analyzer set at 120K resolution with a 1.0 × 10^6^ ion count target. Tandem MS was performed by isolation of the precursor ions with the quadrupole, HCD, and CID fragmentation with a normalized collision energy of 28–30%, and MS^2^ scan analysis with the ion trap set to normal scan rate with an AGC target of 5.0 × 10^5^. Only those precursor ions with charge states of 2–7 were sampled for MS^2^ analysis. The dynamic exclusion duration was set to 10 sec with a 10 ppm tolerance around the selected precursor ion and its isotopes. Monoisotopic precursor selection was turned on. For analysis of tryptic peptides, 250 ng of the digested sample was injected by a Dionex Ultimate 3000 autosampler connected with an Easy-Spray PepMap™ RSLC *C*-18 column (75 μm ID × 15 cm, packed with 3 μm, 100 Å C18 particles). Samples were loaded onto the column for 10 min at 0.300 μL/min. Mobile phase solution A was composed of water and 0.1% formic acid. Mobile phase solution B was composed of 100% acetonitrile and 0.1% formic acid. Mobile phase B solution increases to 2% in the first 5 min, 2% to 22% for 70 min, 22% to 38% for 25 min, 38% to 95% for 15 min, then washed with 95% for 5 min, then back to 2% for 25 min prior to injection of a new sample. Each chromatographic run took 140 min.

### 3.4. Proteomic Data Analysis

The raw mass spectrometry data were processed using Proteome Discoverer (version 2.4, Thermo Fisher Scientific). MS^2^ spectra were searched with a SEQUEST search engine against a customized protein database of *L. hesperus* and UniProt arachnid database (downloaded 3 March 2021). Theoretical peptides were generated from tryptic digestion with up to two missed cleavage events, and carbamidomethylation of cysteine and oxidation of methionine were treated as variable modifications. Precursor mass tolerance was set to 10 ppm and fragment mass tolerance was set to 0.6 Da. Peptide spectral matches were validated using a percolator based on q-values set at a 1% FDR.

### 3.5. Cloning of ECP3 cDNA

One of the peptide sequences SQGNVAMETS SSQAGYGQGQ SYSSNYAATG DSGTGQGGYS SMR, which was identified by our proteomic analyses of tubuliform glands, dope, and egg sacs, was found to have a 100% match to a translated cDNA deposited in the GenBank database with the accession code ADV40223. This cDNA sequence was previously deposited by our laboratory while performing shot-gun DNA sequencing of cDNAs that were randomly selected from a composite cDNA library generated from the seven different silk-producing glands of female black widow spiders. Using this cDNA sequence, we designed primers to amplify the entire coding region of the cDNA, which we later classified as ECP3. The amplified ECP3 cDNA was ligated into the pBAD-Topo cloning vector, and we used this as a template to validate primers used for real-time RT-qPCR analysis.

### 3.6. Real-Time Quantitative PCR Analysis (RT-qPCR)

Reverse transcription reactions were used for real-time quantitative PCR analysis using the DyNAmo HS SYBR Green qPCR kit according to the manufacturer’s instructions. Real-time PCR fluorescence was monitored using an Opticon II thermal cycler (MJ Research Inc.). Amplification products were monitored by SYBR Green detection and routinely checked using melting dissociation curve software and agarose gel electrophoresis. Oligonucleotides used for the analysis of ECP3 expression were the forward and reverse primers 5′-GAA TTC GAG TCA ATG TCC TCG TCT TGC ACT-3′ and 5′-GTC GAC TCA TCG TCG TCC GAA AAC AAA TCC-3′, respectively. ECP2 forward and reverse primers were 5′-CGA AGT GGC AGA ATT TCA ACA TCT G-3′ and 5′-GAA TTG ATT CCA CCG CCT TGA GTG-3′, respectively, while forward and reverse beta-actin primers, which were used for normalization, were 5′-CCC TGA GAG GAA GTA CTC CGT-3′ and 5′-ATC CAC ATC TGC TGG AAG GTG-3, respectively.

## 4. Conclusions

We identified seven proteins that are common to tubuliform glands, its spinning dope, and its product, tubuliform silk. Two of these proteins were uncharacterized molecules previously identified in tubuliform glands as SST proteins, but those proteomic studies did not investigate the tubuliform spinning dope or egg cases [5]. Thus, it was unclear whether these uncharacterized proteins participated in the silk assembly process or were secreted as components of tubuliform silk. Here, we demonstrate using an in-depth proteomic workflow that these two proteins are secreted into the spinning dope of tubuliform glands, and they are extruded with ECP1, ECP2, and TuSp1 as components of tubuliform silk. One of these proteins, ECP3, represents an 11.8 kDa protein, while the other transcriptome scaffold ID #129 predicts a protein with a molecular mass of 113.9 kDa. Intriguingly, this 113.9 kDa protein shares no homology to other proteins deposited within the nrNCBI protein database, warranting further investigation.

Searches of the nrNCBI protein database against the ECP3 protein sequence with the BLASTp algorithm revealed a top match of 45.9% identity to an uncharacterized protein from *Parasteatoda tepidariorum*, the common house spider, with an E-value of 6.0 × 10^−18^. Several other uncharacterized proteins from other spider species are also found within the protein database with slightly larger E-values, suggesting ECP3-like proteins could be potentially present in tubuliform silk spun from other species. The detection of CRP proteins in tubuliform silk, specifically CRP4, also represents the first demonstration that CRP proteins are spun into non-dragline silk fibers. This supports the assertion that CRP family members have a broader role, perhaps a somewhat universal role, in other spider silk types. Although further biochemical research will need to investigate whether CRPs are spun into other fiber types, transcriptome studies support the expression of CRP mRNAs in all silk-producing glands [27].

Our studies provide further insight into the molecular ingredients of tubuliform silk. We provide evidence that tubuliform silk contains at least seven distinct proteins, including TuSp1, ECP1, ECP2, ECP3, CRP4, fasciclin, and translated scaffold ID #129. Additionally, our data, along with other studies, support that MaSp proteins are also components of tubuliform silk [5]. Therefore, it appears the different silk types are composites of large molecular weight spidroins and a handful of low molecular weight proteins. In summary, our findings will further assist in the manufacturing of synthetic silk for future applications.

## Data Availability

Data supporting the reported results will be available from the corresponding author.

## Supplementary Materials

The following are available online, File S1: Proteomic data for egg cases, File S2: Proteomic data for tubuliform glands, File S3: Proteomic data for liquid spinning dope of the tubuliform gland, File S4: Protein sequences of SSTs.

## Sample Availability

Not available.
